# The Economics of Regulating New Plant Breeding Technologies - Implications for the Bioeconomy Illustrated by a Survey Among Dutch Plant Breeders

**DOI:** 10.3389/fpls.2019.01597

**Published:** 2019-12-20

**Authors:** Justus Wesseler, Hidde Politiek, David Zilberman

**Affiliations:** ^1^Agricultural Economics and Rural Policy Group, Wageningen University & Research, Wageningen, Netherlands; ^2^Department of Agricultural and Resource Economics, University of California, Berkeley, Berkeley, CA, United States

**Keywords:** CRISPR-Cas, regulation, plant breeding sector, impact, Dutch plant breeders, Court of Justice of the European Union, genetically modified organism, new plant breeding technologies

## Abstract

New plant breeding technologies (NPBTs) are increasingly used for developing new plants with novel traits. The science tells us that those plants in general are as safe as than those once developed using “conventional” plant breeding methods. The knowledge about the induced changes and properties of the new plants by using NPBTs is more precise. This should lead to the conclusion that plants developed using NPBTs should not be regulated differently than those developed using “conventional” plant breeding methods. This contribution discusses the economics of regulating new plant breeding technologies. We first develop the theoretical model and elaborate on the different regulatory approaches being used and compare their advantages and disadvantages. Then we provide a perspectives on EU regulation around mutagenesis-based New Plant Breeding Techniques (NPBT), formed by new insights from a survey among Dutch plant breeding companies. The survey measures the attitude of breeding companies towards the ruling of the EU Court of Justice that subjected the use of CRISPR-Cas in the development of new plant varieties under the general EU regulations around GMOs. The results show that plant breeders experience a financial barrier because of the ruling, with perceived negative impact on competitiveness and investments in CRISPR-Cas as a result. The degree of negative impact differs however significantly among seed-sectors and company sizes. One of the most striking results was the relative optimism of companies in the sector about more lenient legislation in the next five years, despite the stated negative effects.

## Introduction

The design of a regulatory regime for new plant breeding technologies (NPBTs) is under discussion in the European Union (EU), the United States (US), Canada, and many other parts of the world. In particular, it is being discussed whether or not they should be regulated similarly to genetically modified organisms (GMOS) or non-GMOs, or whether they need special regulations (Eriksson et al., 2019). In the case of the EU, since NPBTs include a wide range of methods, some applications will result in crops to be considered a GMO under the EU regulatory system, and others not (see Sprink et al., 2016 for an overview). Even if they are considered to be a GMO, simplified approval processes might be possible (Purnhagen et al., 2018).

The EU policies on NPBTs will have implications for international trade and regulatory systems in other countries (Wesseler et al., 2017), and vice versa. Further, regulatory approaches affect the duration and cost of the approval process with related implications for investments in plant breeding (Kalaitzandonakes et al., 2007; Smart et al., 2017; Smyth et al., 2017).

Stringent regulations of GMOs have impacts expanding beyond agriculture. It is often presumed that the stringent regulations of GMOs in the EU only affect the agriculture and food sector, but not the medical sector and other parts of the bioeconomy. While this line of reasoning may apply to consumer attitudes toward biotechnology, it is misleading in a broader context. There is some evidence of negative spill-overs of the presumed stringent regulations on clusters of regularly interspaced short palindromic repeat (CRISPR)-based technologies in the EU to the medical as well as other sectors. A recently published survey (Martin-Laffon, 2019) on the CRISPR-patent landscape shows patent applications in the EU are substantially lacking behind other regions in the world, and not only in agriculture but also in the fields of medical, industrial, and technical applications (**Table 1**).

**Table 1:** Number of CRISPR Patent Families by Technical Fields and EU Share.

Technical field	Total number	European Union	
		No.	%
Agricultural	374	18	4.8
Industrial	192	23	12.0
Medical	614	19	3.1
Technical improvement	1,052	76	7.7

Source: based on data published in Martin-Laffon et al. (2019). Regional identification of patent applications has been done by first priority date.

In this contribution the economics of regulating NPBTs and their implications are presented and discussed. First, a general economic model of regulation and its implications for investment in NPBTs is introduced, followed by a presentation of the potential implications based on a recent survey of the Dutch plant breeding sector.

### The Economics of Regulating New Plant Breeding Technologies

The demand for regulating NPBTs originates from concerns about potential negative implications for human health and the environment. There are two strategies for regulation that can be combined. One is imposing *ex-ante* regulatory standards for prior approval before commercialization. Second is *ex-post* liability rules to compensate for damages and to penalize non-compliance with *ex-ante* regulations. Economic research analyzes the mixture of these two regulatory approaches (see e.g., Kolstad et al., 1990).

The advantage of *ex-ante* regulatory standards is that potential damage can be reduced before damage actually happens. The disadvantage is that those standards apply uniformly without recognizing heterogeneity among applications as well as applicants. Some applications and/or applicants might be over-regulated while others might be under-regulated (e.g., Shavell, 1984). The main problem for *ex-ante* regulatory standards is caused by information asymmetries between the regulator and the firm. The firm has more detailed knowledge about the product than the regulator. Firms in general are required to provide a set of standard regulatory information, but regulators have the option to seek additional case-specific information. This option can increase the cost and delay approval. The approval process may entail performance standards that have to be followed, which can in some cases be prohibitively high, such as in the case of some GMO coexistence regulations (Beckmann et al., 2010).

*Ex-post* liability applies when companies face legal challenges from externalities of e.g., NPBTs, such as health or environmental claims. Those threats provide incentives for companies to take *ex-ante* voluntary precautions to address potential health and environmental safety issues. Shleifer (2010) notes that perfect *ex-post* liability regulations would be sufficient to ensure that users of NPBTs do not expose themselves to liability greater than the damage costs they would face. If the penalties correspond to social costs, then its outcomes are optimal, which is consistent with Coase (1960). However, a perfect system requires that the damage and the liable party can be correctly identified and that juries are not corrupt or biased. When this is not the case, because of imperfect information and financial considerations, a policy combining *ex-ante* regulatory standards and *ex-post* liability systems can improve social welfare (Kolstad et al., 1990). The challenge is to identify the right combination of *ex-ante* regulatory standards and *ex-post* liability rules.

The incentive for firms to invest in NPBTs largely depends on the net benefits of the investment, which are influenced by *ex-ante* regulatory standards and *ex-post* liability. In making an investment decision, the product life can be divided in four important phases: research and development (R&D); approval (A); marketing (M); post-marketing liability (L). All these phases are characterized by uncertainty over the benefits and costs as well as by their time length (see e.g., Purnhagen and Wesseler, 2019).

The R&D phase includes multiple uncertainties including the probability of success and the time taken to obtain it, the costs of testing new ideas, as well as upscaling them. These costs are affected by regulation, as compliance with regulations may extend the duration of research in the lab and the field, and increase the costs. Some countries even have strict field trial requirements that are often technically infeasible and economically unviable (Kuntz, 2012). The requirement to publicize the location of field trials in some EU member states, e.g., has resulted in public protests making it almost impossible for companies to conduct those trials.

The approval process can also add substantial costs. Research shows that the direct costs of the approval process varies substantially, from a few thousand USD to several millions (Smyth et al., 2017), depending on the regulatory environment, while the time length of the approval process also varies widely (Jin et al., 2019). The time length of the approval process adds additional costs for plant breeders as it delays market access, but also for other participants in the food and feed supply chain, caused by the asynchronicity in approval at country level affecting international trade (European Commission, 2007; Backus et al., 2009).

The production and marketing of approved products also faces regulations. The coexistence regulations in the EU may severely limit where GMO traits can be produced and thus increase production costs. Food and feed products derived from the use of NPBTs also need to comply, as other food and feed products, with the EU laws on food and feed (Purnhagen, 2019). Furthermore, many countries have implemented labeling policies for GMOs that make GMOs differentiated products from non-GMOs (Castellari et al., 2018). This poses an additional challenge when there is no detectable difference between NPBTs and “conventional” products for international trade, product differentiation *via* labeling, and coexistence.

In summary, the regulatory environment effects the costs and benefits of investments in NPBTs. As the regulatory environment differs by country and region, this provides different incentives for plant breeders for their choice of investments.

### Economic Implications of Regulation and Delayed Approvals for Plant Breeding: The Case of the European Union

In the summer of 2018, the Court of Justice of the EU ruled that on CRISPR-based plant breeding technologies are not immediately exempted from existing EU regulation of GMOs (Purnhagen et al., 2018). The ruling frustrated many in the field of biotechnology and led experts to speculate about its potential effects on EU-based plant breeders. These concerns relate to the cost of approval procedures and their potential negative impact on competitiveness and firms’ investments in CRISPR-based technologies (Callaway, 2018). They include wider implications for food security (Zaidi et al., 2019) and the development of the bioeconomy, particularly in Europe (Wesseler and von Braun, 2017).

In the EU, cultivation of genetically modified plants is nearly negligible due to procedures required for bringing plants classified as GMOs to market. In 2017, only 131,535 (James, 2017) of the 11.9 million total hectares for permanent crops (Eurostat, 2018) were planted with the one genetically modified crop approved for cultivation, an insect-resistant maize. It was expected that the introduction of new more precise and nature-like plant breeding techniques, especially CRISPR-Cas, would overcome the resistance to the application of modern biotechnology in plant breeding and unleash the potential of improved plant varieties (Eriksson, 2019). The recent ruling by the EU’s highest court that requires plants developed by these mutagenesis-based modification methods to follow the approval process for GMOs, therefore came as a blow (Purnhagen et al., 2019). First of all, the theoretical model suggests the EU GMO approval procedure to be a major barrier for the use of CRISPR-Cas in plant breeding. Besides that, it is expected that the nature of the approval procedure has negative consequences for the investments in CRISPR-Cas technique. Furthermore, one can expect the decision to lead to competitive disadvantage for plant breeding companies. A recent survey of Dutch plant breeding companies gives a first empirical insight on the impact of the ruling.

Among the EU-members, the Netherlands has an especially strong position in the development, propagation and trade of reproduction materials. Around 40% of all globally traded vegetable seeds and 60% of traded seed potatoes are of Dutch origin. The Dutch seed sector also contributes 60% of applications for plant breeder rights (Government of the Netherlands, 2017), making it a core location for the development of plant reproduction materials in the EU. These characteristics make the sector a sensible object of study for a first assessment of the expected effects and implications of the ruling.

## Materials and Methods

The population we consider consists of companies that are affiliated with PLANTUM, the association that serves the interests of around 350 companies in the plant breeding sector in the Netherlands. In 2011, of the then 400 Dutch companies active in the plant breeding sector, 385 were affiliated with PLANTUM (Kokcis et al., 2013). The high coverage of the PLANTUM database shows that this population includes 87.5 per cent of Dutch plant breeding companies.

To judge the representativeness of the sample, usually a description of the population should be sketched. Unfortunately, no quantitative overview exists of the Dutch plant breeding sector, categorized by seed sector. However, considering the former high coverage ratio of the PLANTUM company database, the population we consider can be assumed to cover a vast majority of the population. Within the online company database the companies which are categorized under “agriculture” (31 units), “fruit trees” (1 unit), “*in vitro laboratoria*” (9 units), “vegetable seeds” (25 units), and a selection of the relevant actors (9 out of 22) within “other services” have been considered to be part of the population we consider. This led to a population of 75 units. Due to the absence of contact details, three units had to be removed (all in “agriculture”) resulting in a sampling population of 72 (**Table 2**).

**Table 2:** Distribution of the population (sample) across the defined categories of seed sectors and company sizes.

	Seed potato	Vegetables	Agriculture	Fruit trees	*In vitro* Labs	Other	Multi	Total	Share (%)
Micro (<10 empl.)	2 (3)	3 (1)	1 (0)			2 (0)	1 (1)	9 (5)	13 (15)
Small (10 to 49 empl.)	3 (1)	10 (4)	4 (2)	1 (0)	5 (0)	3 (2)	1 (0)	27 (9)	38 (27)
Medium (50 to 249 empl.)	1 (1)	4 (2)	2 (2)		2 (2)	3 (0)	0 (2)	12 (9)	17 (27)
Large (250+ empl.)	4 (2)	8 (5)	8 (3)		2 (0)	1 (0)	1 (0)	24 (10)	33 (30)
Total	10 (7)	25 (12)	15 (7)	1 (0)	9 (2)	9 (2)	3 (3)	72 (33)	
Share (%)	14 (21)	35 (36)	21 (21)	1 (0)	13 (6)	13 (6)	4 (9)		100 (100)

Categorization is based on company profile as publicly provided by the companies. Company-size categories as defined in EU recommendation 2003/361. Differences in company sizes are the result of the categorization by companies themselves, as reported in brackets, as opposed to the categorization based on publicly available information.

The online survey was distributed by e-mail to the general contact address of the company. The guiding text asked to forward the survey to the relevant R&D manager within the company. Whenever the address was available, the survey was directly sent to the relevant R&D manager or department. The survey was open for response from 7th to 20th February 2019. During the course of the survey, two reminders were sent to the units in the sample.

**Figure 1:**
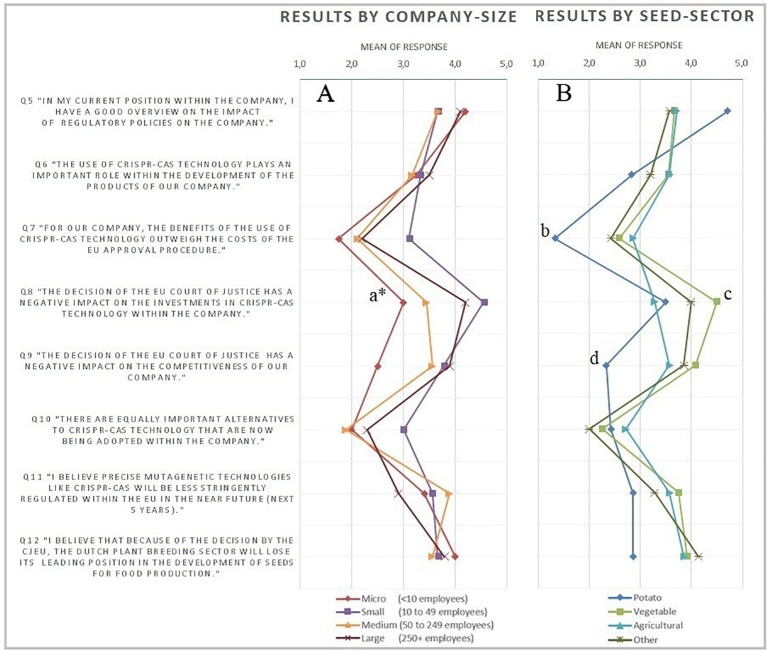
Overview of average results of the survey statements on a five-point Likert-scale, excluding *not relevant (0)* responses ranging from (1) *strongly disagree* to (5) *strongly agree*. Graph panel **(A)** gives the results differentiated by company size, whereas panel **(B)** differentiates the results on seed sector. Statistical results graph panel **(A)**: a* = two-sided significant difference micro from rest (P < 0.05) and two-sided significant different distributions among all groups (P < 0.05). Statistical results graph panel **(B)**: b = two-sided significant difference potato from all (P < 0.05). c = two-sided significant difference vegetable from all (P < 0.05). d = two-sided significant different potato from all (P < 0.05).

The survey starts with four questions on the profile of the company in terms of size, seed sector, country of headquarter and main market. The number of employees was chosen as the measure of company size, since it is expected to lead to more reliable data than sales. The European Commission (EC) definition of company size, defined in EU Recommendation 2003/361, was followed. The respondents were also requested to state their understanding of the impact of regulation on the company. This way, any severe bias due to a respondent’s lack of knowledge could be identified and possibly corrected, *ex post*. The body of the survey consisted of eight questions about: 1) the respondents overview on the impact of regulatory policies on the company; 2) the role of CRISPR-Cas within the company; 3) the effects of the structure of the current GMO regulatory framework on investment in CRISPR-Cas; the impact of the recent court ruling on 4) the investments and 5) the competitiveness of the company; 6) the use of alternative technologies; 7) the prospects of changes in EU legislation and 8) its effects on the Dutch plant breeding sector. These questions were answered on a five-point Likert-scaled range of response possibilities, ranging from *strongly disagree* to *strongly agree*. For some questions a *not relevant*-option was provided. Lastly, there was room for additional comments. The Likert-scale is one of the most used and reliable scales to measure opinions and underlying motives of behavior (Burns and Bush, 2008). Nevertheless, the correct way to deal with the data resulting from surveys with a Likert-type scale has often been debated. Specifically, the disagreement addresses whether resulting data should be dealt with as ordinal or cardinal measurements. This has implications for the statistical methods that need to be used in the analysis of the data. Although it is rather customary to deal with such data as being cardinal, the intervals cannot be assumed to be equal from a theoretical perspective (Sullivan and Artino, 2013). Considering this, and further assumptions on the structure of the data (e.g., sample size and normality), in this research it seems better justified to regard the data as being ordinal for statistical analysis. Therefore, non-parametric tests will be applied. However, it should be noted that averages will be used in the graphical presentation of the data (**Figure 1**). Averages are preferred in the graphical presentation, because they pose less risk for extreme (i.e., misleading) results compared to other central tendencies (e.g., modus). One should, however, consider the ordinal character of the data when directly comparing means of subgroups. An overview of the complete survey and the data is provided in the **Supplementary Materials**.

## Survey Results

As the sample is based on the subjective view of representatives of the firm, it is important to consider the capability of the respondent to form a well-founded opinion on the statements. Therefore, the respondents have been asked to self-assess their knowledge on the impact of regulatory policies on the company. The majority (72.8%) of the respondents agree or strongly agree with the statement of having a good overview on the impact of regulatory policies on their company. Only four (12.1%) of the respondents disagree with this statement. This gives confidence for a high validity of the answers on the core measurements of the survey. Furthermore, the importance of the CRISPR-Cas technology for the companies responding is of interest. About 45% of the respondents agree or strongly agree with the statement *“The use of CRISPR-Cas technology plays an important role within the development of the products of our company*.” In general, the results show no clear tendency in the current importance of use within the companies across the different seed sectors. Also, no significant differences were found when testing on differences across company sizes.

The main results of the survey are provided in **Figure 1**, showing the average scores, differentiated by seed sector in **Figure 1A** and by company size in **Figure 1B**.

**Figure 1** shows the perception of negative effects of the ruling on investments and competitiveness. Micro-sized companies expect significantly milder effects on competition than larger-sized companies. The relatively technology-intensive sector of vegetable seeds development (Kokcis et al., 2013) expects the strongest negative effects on competitiveness and investments in CRISPR-Cas applications. Companies in the relatively technology-extensive seed-potato development have diverse expectations (Q6). While they most strongly believe regulatory costs outweigh the benefits of CRISPR-Cas (b), on average they disagree that the ruling will negatively affect competitiveness. This contrasts with expectations of experts in the field (Van ‘t Hoog, 2019). CRISPR-Cas has especially high potential in speeding up seed-potato development times (Andersson et al., 2018), but breeding companies still expect the bureaucratic hurdle to be too costly. For the potato production in particular this is disappointing as CRISPR-Cas applications are expected to control major diseases that would allow to substantially reduce fungicide use with related benefits for the environment such as reduced environmental and emission of greenhouse gases.

In the survey, Q8 measures the effect of the Court of Justice of the European Union (CJEU) ruling on investments in CRISPR-Cas technology among Dutch plant breeding companies. The vast majority of the respondents *agree* or *strongly agree* (30.3% and 39.4%, respectively) with the statement in Q8: “*The decision of the EU Court of Justice has a negative impact on the investments in CRISPR-Cas technology within the company*.” There appears to be a strong negative effect of the decision of the CJEU on the investments in CRISPR-Cas technology. Looking at **Figure 1A**, the mean responses to statement Q8 appear to differ across company size. The micro- and small-sized companies agree the most on average and experience the least negative impact on investments. The Mann-Whitney U test statistically confirms this difference. The two-tailed (exact) significance is 0.013, which shows that micro-sized companies agree significantly less with the proposed statement in Q8. This is not surprising, as it is intuitively less likely for micro-sized companies to be able to invest in CRISPR-Cas technology anyway. The differentiation in the responses among company sizes is confirmed by the results of the corresponding Kruskal-Wallis test (see **Supplementary Material** for details). Looking at the differences in responses across sectors, mainly the vegetable sector indicates strong negative effects on investments in CRISPR-Cas because of the CJEU ruling. The vegetable sector appeared to agree significantly more with statement Q8 than companies in other sectors. This result has been denoted with letter c in **Figure 1B**.

The third hypothesized effect of the CJEU judgment relates to the comparative disadvantage that plant breeding companies in the Dutch plant breeding sector may be confronted with. This hypothesis was primarily tested by the statement Q9: “*The decision of the EU Court of Justice has a negative impact on the competitiveness of our company*.” A majority of 60.6% of the respondents agreed or strongly agreed with this statement, whereas 24.2% disagreed. Testing any differences between company size and the (negative) effects on competition by means of a Kruskal-Wallis test, provides no significant differences. However, the boxplot diagram in the **Supplementary Material** shows a large difference between the modus of the results on statement Q9 of micro-sized companies compared to other-sized companies, while the Mann-Whitney U test does not confirm significant differences and the result therefore needs to be regarded with care.

Nevertheless, the different effect in competitiveness for the micro-sized companies as compared to larger sized companies might, as suggested by one respondent, be due to the initial accessibility of CRISPR-Cas technique. Smaller, less capital-intensive companies are expected to have lower accessibility to this technique. Therefore, these companies might enjoy some benefit from the ruling of the CJEU, as it equalizes the playing field in terms of use of technology. In the additional comments, a respondent from a micro-sized (<10 employees) remarked: “*We have no possibilities to use these new techniques and therefore can profit slightly from the EU ban on these techniques*.” This level playing field has also been pointed out by an EU market-oriented, large (>250 employees) company as well. One respondent commented for example: “*Only a level playing field in the EU is crucial. End customers will get the products they want. If that is food without mutations, we’re fine with that*.” This would, however, only be true for companies who compete within the EU-market. For EU-based companies who primarily compete on non-EU markets, the limiting factors of the CJEU ruling might lead to larger negative effects in competitiveness. Note that two of the three respondents who indicated that they are moving their research outside the EU, had a non-EU main market. This relation between main-market and (negative) effect on competitiveness could, however, not be confirmed statistically. Based on the Kruskal-Wallis test, no significant difference between the effects on competitiveness across the main market (two-sided asymptotic sig.: 0.170) could be seen. Also, no significant result was found when differentiating between companies with main market within the EU or non-EU (2-sided asymptotic sig, 0.865). Although the latter result was likely to have low significance due to the small number (N = 5) of respondents having a non-EU main market. Besides the strong tendency toward agreement with the statement in results of Q9, three respondents specifically expressed their concerns about the competitiveness for the EU. Most striking is that the companies in the potato sector on average disagree with the statement Q9, and therefore differ from all other sectors. This can also be statistically confirmed by a Mann-Whitney U test. Further analysis among the company characteristics shows that, with one exemption (Africa), all companies in the potato sector appeared to have their main market within the EU. Moreover, the two respondents who pointed out the importance of a level-playing field (over the importance of the use of CRISPR-Cas), were both in the potato sector.

Besides testing the hypothesized negative effects of the CJEU Ruling, the results of the survey allow insights in two more factors that are related to the impact of the hypothesized effects of the ruling of the CJEU. First of all, the effects of the decision depend on the existence of equally important alternatives. When the substitutability of CRISPR-Cas technique is high, any limiting factors of the EU GMO procedure might be diminished. The response to the statement (Q10): “*There are equally important alternatives to CRISPR-Cas technology that are now being adopted within the company*,” indicates that the substitutability of CRISPR-Cas appears to be rather low. Only 15.2% of the respondents agreed on having equally important technologies adopted within the company. Especially *in vitro* labs and companies who operate in multiple sectors (including operations as *in vitro* lab) appear to disagree with statement Q10.

Surprisingly, there appears to be a relatively positive attitude of the respondents toward the prospects of the strictness of the EU legislation around mutagenesis-based NPBTs. A majority of 60.6% of the respondents agreed to some degree with the statement in Q11: “*I believe precise mutagenetic technologies like CRISPR-Cas will be less stringently regulated within the EU in the near future (next 5 years)*.” When looking at the differences in company sizes (**Figure 1A**), large companies appear to have a relative more pessimistic view, compared to smaller-sized companies. However, no significant differences were found. Among the seed sectors (**Figure 1B**), companies operating in the potato sector are relatively pessimistic, as compared to the other sectors. However, also no significant difference was found.

This general positivism about the development of legislation in the near future (within the next 5 years), is somewhat contrary to the pessimism when asking about the position of the Dutch plant breeding sector as a world leader in the development of seeds for food production. The majority of 63.6% agreed with the statement (Q12) that the Dutch plant breeding sector will lose its leading position in the development of seeds for food production. There seems to be a strong consensus independent of company size (**Figure 1A**). The same applies when differentiating the results by seed sector (**Figure 1B**), although companies in the potato sector appear to be relatively less pessimistic.

## Discussion and Conclusions

The decision of the CJEU currently places plants produced by NPBTs under the regulations for GMOs. This includes approval and marketing costs, and may result in disincentives for investment in NPBTs in particular in the EU. The survey of Dutch plant breeding companies largely confirms this intuition. The survey also shows that companies with markets outside the EU intend to reallocate their research. Companies that mainly serve the European market and are smaller in size expect their competitiveness to be less affected by the ruling. Nevertheless, the companies agree that the decision will have negative implications for the competitiveness of the Dutch plant breeding sector. Surprisingly, the companies are very optimistic that mutagenetic plant breeding technologies like CRISPR-Cas will be less strongly regulated in the near future.

There is some support for this optimism. A number of stakeholder groups have urged the European Commission to update the approval process for GMOs. A citizens initiative launched by students (https://eci.ec.europa.eu/011/public/#/screen/home) asks for the development of a list of plant breeding technologies that will be exempted from the Directive 2001/18 and would not be considered as GMOs. This is a sensible approach, as it would also avoid the need for labeling products and related problems derived from plants developed by exempted NPBTs. The process for changing the Directive 2001/18 will be difficult. EU member states hold deeply entrenched and diverging views on GMOs (Smart et al., 2015), and finding a qualified majority for a change will remain a challenge for the European Commission. The results of the survey presented and the results on the patent landscape for CRISPR illustrate the importance and urgency for a change if the EU does not want to fall any further behind in the development and use of the technology. They also suggest negative implications for African development and adaptation to climate change (Wesseler et al., 2017).

Regulating NPBTs similar to “conventional” breeding technologies does not imply that food products will not be regulated. In the EU, food products will still be regulated under the EU food law (Purnhagen, 2019). The same can be observed for other countries (Eriksson et al., 2019). If NPBTs do not fall under the GMO regulation, labeling for food products will be simplified and it reduces costs. A voluntary market for negative labeling in the form of “does not contain…” may emerge similarly to what has been observed in the case of GMOs in the US and the EU (e.g., Castellari et al., 2018; Venus et al., 2018). The advantage of such a labeling scheme is that it is a market-driven response to a demand among some consumers, and similar to products sold under an organic label. Companies participating in such a labeling scheme do this at their own risk and can even differentiate their products according to the labeling schemes.

## Author Contributions

JW wrote major parts of the contribution, supervised the survey, and finalized the contribution. HP conducted the survey and wrote major parts of the survey results. DZ contributed to the section on the economics of NPBTs and the *Introduction* and *Discussion and Conclusions* sections and reviewed the complete contribution.

## Conflict of Interest

The authors declare that the research was conducted in the absence of any commercial or financial relationships that could be construed as a potential conflict of interest.
