# Supplementary material for: The Economics of Regulating New Plant Breeding Technologies - Implications for the Bioeconomy Illustrated by a Survey Among Dutch Plant Breeders
Source: Front Plant Sci. 2019 Dec 20;10:1597. doi: 10.3389/fpls.2019.01597 (PMC6932994; doi:10.3389/fpls.2019.01597)
Supplement: Supplementary file 1 [file DataSheet_1.docx]

Supplementary Material

# Detailed Results

The results from the survey can help to gain empirical insight in the hypothesized effects of the decision by the CJEU for the Dutch plant breeding sector. First the descriptive statistics of the survey will be presented, followed by the core observations and statistical findings. These findings will directly be confronted with the three hypotheses. Besides that, surrounding moderating factors for the (hypothesized) effects like the level of substitutability of CRISPR-Cas technique and the expectations on future legislation will be discussed.

*Descriptive statistics*

Of the 72 units in the sample, 33 responded to the sample, yielding a response rate of 45,8%. The sample units were distributed across the seed sectors as provided in table 1.

Table 1 Distribution of responding units across the defined categories of seed-sectors and company-sizes. This distribution is the result of the own answers of respondents to the survey.

|  | **Seed-Potato** | **Vegetables** | **Agriculture** | **Fruit trees** | **In Vitro Labs** | **Other** | **Multi** | **Total** | **%** |
| --- | --- | --- | --- | --- | --- | --- | --- | --- | --- |
| Micro (<10 employees) | 3 | 1 |  |  |  |  | 1 | **5** | **15,1%** |
| Small (10 to 49 empl.) | 1 | 4 | 2 |  |  | 2 |  | **9** | **27,3%** |
| Medium (50 to 249 empl.) | 1 | 2 | 2 |  | 2 |  | 2 | **9** | **27,3%** |
| Large (250+ employees) | 2 | 5 | 3 |  |  |  |  | **10** | **30,3%** |
| **Total** | **7** | **12** | **7** | **0** | **2** | **2** | **3** | **33** |  |
| **%** | **21%** | **36%** | **21%** | **0%** | **6%** | **6%** | **9%** |  | **100%** |

Table 2 Distribution of sample units across the defined categories of seed-sectors and company-sizes. Categorization is based on desk research.

|  | **Seed-Potato** | **Vegetables** | **Agriculture** | **Fruit trees** | **In Vitro Labs** | **Other** | **Multi** | **Total** | **%** |
| --- | --- | --- | --- | --- | --- | --- | --- | --- | --- |
| Micro (<10 employees) | 2 | 3 | 1 |  |  | 2 | 1 | **9** | **13%** |
| Small (10 to 49 empl.) | 3 | 10 | 4 | 1 | 5 | 3 | 1 | **27** | **38%** |
| Medium (50 to 249 empl.) | 1 | 4 | 2 |  | 2 | 3 |  | **12** | **17%** |
| Large (250+ employees) | 4 | 8 | 8 |  | 2 | 1 | 1 | **24** | **33%** |
| **Total** | **10** | **25** | **15** | **1** | **9** | **9** | **3** | **72** |  |
| **%** | **14%** | **35%** | **21%** | **1%** | **13%** | **13%** | **4%** |  | **100%** |

Table 2 has been compiled based on desk-research of the sample units, meaning through calling the contacts, or information provided on the company website. Overall, the distribution of the respondents across the seed sectors is comparable to that in the initial sample (see Table 2). However, there appears to be a slight overrepresentation of the respondents in the (seed-)potato and vegetables sector (comparing to the initial distribution of the sample in Table 2). Also, the In Vitro-labs are slightly underrepresented in the sample. However, all the 3 respondents who selected multiple seed sectors, also included “In Vitro Lab”. In terms of company-size, there appears to be some over-representation of the medium sides companies amongst de respondents, compared to the initial sample (23,3% against 17% in the sample). In general, the distribution of characteristics of the sample gives a good basis for making distinctions between the effects between company sizes and seed-sectors within the analysis for the hypothesized effect. For clarity of the visual presentation, the grouping of the seed-sectors has been limited to 4 for the Frontiers Perspective. Also, no sensible statistical analysis could be made based on the separate groups of “fruit trees”, “In Vitro Lab” and “Other”, due to their limited number of units, which therefore have been grouped to (“other”). Table 3 further shows the raw responses to the descriptive questions of the survey. In terms of market orientation, the main focus lies on The Netherlands (36,4%) and the rest of the EU (48,5%). Also, the clear majority (91,0%) of the companies that responded have their headquarters based in the Netherlands.

**Table 3.** Descriptive statistics of responding sample units (see survey).

As the sample is based on the subjective vision of representatives of the firm, it is important to further gain insights in the capability of the respondent to form a well-founded opinion on the statements. Therefore, the respondents have been asked to self-assess their knowledge on the impact of regulatory policies on the company. Table 4 provides an overview of the responses on the corresponding statement (Q5).

Table 4. Distributions on self-assessment in overview on impact of regulatory policies on the company.

The majority (72,8%) of the respondents agree or strongly agree in the statement of having a good overview on the impact of regulatory policies. Only 4 (12,1%) of the respondents disagree with this statement. This gives confidence for a high validity of the answers on the core measurements of the survey, which will be presented in the main results.

It is furthermore important to consider the role of CRISPR-Cas technology for these companies in the initial situation. The statement in Q6 has aimed to measure this (see Table 4).

Table 5. Distribution of responses on statement Q6: "The use of CRISPR-Cas technology plays an important role within the development of the products of our company."

In general, the results show no clear tendency in the current importance of use within the companies across the different seed sectors. The results of a Kruskal-Wallis test for the differences confirms this. From the test can be concluded that, in general, no significant differences (asymp. sig.=0,325) exist within the results on Q6 among the different seed-sectors. Also, no significant differences were found when testing on differences across company sizes (asymp. sig.=0,353).

*Main Results*

For the testing in the initial analysis of the survey, three hypotheses had been posed, based on expert statements. First of all, experts expect the EU GMO approval procedure to be a major barrier for the use of CRISPR-Cas in (edible) plant breeding for Dutch plant breeding companies (H1). Besides that, it is expected that the nature of the approval procedure has negative consequences for the investments in CRISPR-Cas technique within the Dutch plant breeding companies (H2). Furthermore, it is feared that the decision will lead to competitive disadvantage for the concerned Dutch companies (H3).

The first hypothesized effect of the decision by the CJEU is the limiting effect of the EU GMO approval procedure for the use of CRISPR-Cas-technique in the development of new plant varieties. In the survey, this was measured by statement Q7: *Please indicate how strong you agree with the following statement: "For our company, the benefits of the use of CRISPR-Cas technology outweigh the costs of the EU approval procedure "*. The results show that a majority (63,7%) of the respondents disagree in some extent with the statement made in Q7. Figure 1A (figure 1, panel A) shows a relatively strong consensus towards disagreement by micro, medium and large-sized companies. Figure 1B of the main text shows that companies in the (seed-)potato sector appear to respond specifically negative to Q7. A Mann-Whitney U test showed the distribution of responses on Q7 of companies in the potato-sector to differ significantly from companies in the other sectors (Exact sig= 0,021). This statistical difference has been denoted with letter b in the figure 1, panel B. The statistical output of this test is provided in figure A.

Table 6. Distribution of answers on statement Q7: "For our company, the benefits of the use of CRISPR-Cas technology outweigh the costs of the EU approval procedure."

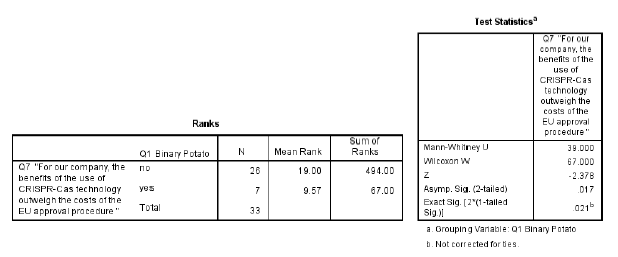


Figure A. Output of Mann-Whitney U test on difference between companies in (seed-)potato sector and all other companies.

Further statistical analysis shows however that there are no significant differences after categorizing in the results based on the size of the company or the seed-sector in general. The Kruskal-Wallis H Test gives a significance level of 0.117 for the test on differences in results across company size. In general, no statistical proven difference exists between the company-sizes in terms of the way they experience a barrier by the EU approval procedure. A similar result was found when grouping on seed-sector and performing a Kruskal-Wallis H test. This resulted in an asymptotic significance of 0,091, which is lower than the (standard) significance level of 0,05. Therefore, it is not shown that, in general, there exist significant difference in the results on Q7 across the seed sectors.

In the survey, Q8 measures the effect of the CJEU ruling on investments in CRISPR-Cas technology among Dutch plant breeding companies., again through a statement. The statement was as follows: "*The decision of the EU Court of Justice has a negative impact on the investments in CRISPR-Cas technology within the company*". The vast majority of the respondents *agree* or *strongly agree* (30,3% and 39,4%, respectively) with the statement in Q8. Therefore, there appears to be a strong negative effect of the decision of the CJEU on the investments in CRISPR-Cas technology. Looking at Figure 1A in the main text, the mean responses to statement Q8 appear to differ across the company sizes. The micro- and small-sized companies agree the most on average. Apart from that, larger sized companies appear to have a higher level of agreement on average. Looking at the differences in responses across company-sizes, mainly the vegetable sector appears to experience strong negative effects in investments in CRISPR-Cas because of the CJEU ruling. The differentiation in the responses among company-sizes is confirmed by the results of the corresponding Kruskal-Wallis test. Output of the Kruskal-Wallis test on differences across company-sizes, is provided in Figure B below.


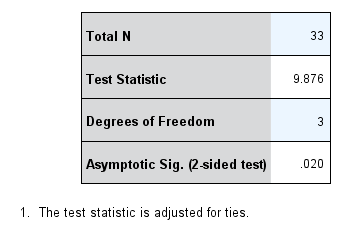

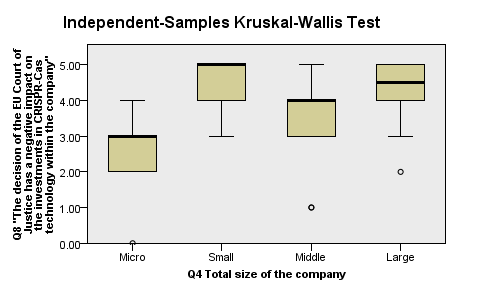


Figure B. Box-plot and results of Kruskal-Wallis test on response on Q8, categorized by total company size.

The results of the test (Figure B) show that, in general, significant differences exists between the results of the Q8 across company size. This result has been denoted with letter a* in figure 1, panel A of the main Frontiers submission. Looking at the individual differences, it becomes clear that micro-sized companies appear to experience the least negative impact on investments. The Mann-Whitney U test statistically shows this difference. The two-tailed (exact) significance is 0,013, which shows that micro-sized companies agree significantly less on the proposed statement in Q8 (results in figure C). This is not surprising, as it is intuitively less likely for micro-sized companies to be able to invest in CRISPR-Cas technology anyway. This relation between investments and importance of use of CRISPR-Cas can however not be statistically proven based on test with the results on Q6. Also, no significant (asymptotic sig=0,056) result was found when testing on general differences across seed sectors (Kruskal-Wallis H test). However, the vegetable sector appeared to agree significantly (exact sig=0,040) more to statement Q8, than companies in other sectors. The results of this test are provided in Figure D. This result has been denoted with letter c in figure 1, panel B of the main submission

.


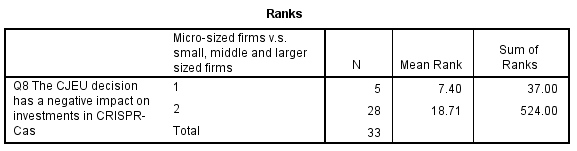

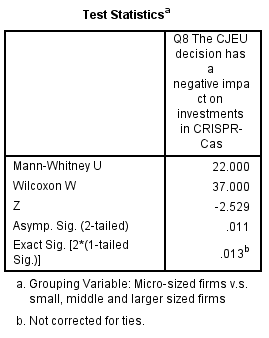


**Figure C**. Results of Mann-Whitney test on differences on Q8 between micro-sized firms and the other firms.


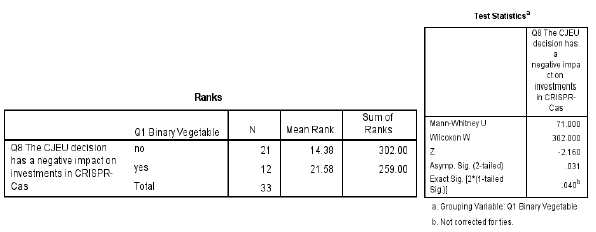


**Figure D.** Statistical output of Mann-Whitney U test, grouping on vegetable seed-sector and non-vegetable seed-sector.

The additional comments on the survey of the companies listed below gives some more insight in the impact on investments in CRISPR-Cas. Respondents 5, 22 and 29 commented that they are moving research locations out of the EU because of the ruling. This means that these individual companies do not necessarily reduce their investment in CRISPR-Cas technology, but are even willing to relocate research to further invest in the technology. Although all three companies have their headquarters based in the Netherlands, two companies have a non-EU main market. Notable is also, that all three companies operate (among others) as In Vitro lab.

The third and last hypothesized effect of the CJEU judgment relates to the comparative disadvantage that plant breeding companies in the Dutch plant breeding sector may be confronted with. This hypothesis was primarily tested by the statement Q9: *"The decision of the EU Court of Justice has a negative impact on the competitiveness of our company".* A majority of 60,6% of the respondents agreed or strongly agreed with this statement, whereas 24,2% disagreed. Therefore, the expected effects of the hypothesis are confirmed by the results on first sight. However, further analysis can be done on the influence of the of the company size and main market within these results. In the additional comments, a respondent (27) from a micro-sized (<10 employees) remarked: “*We have no possibilities to use these new tecniques and therefor can profit slightly from the EU ban on these techniques*”. This remark implicates the role of a differentiation in effects in terms of the financial means of a company. Most logically, this is related to the size of the company. Figure E gives the output to a Kruskal-Wallis test on company size.

Table 7. Distribution of answers on statement Q9: "The decision of the EU Court of Justice has a negative impact on the competitiveness of our company."

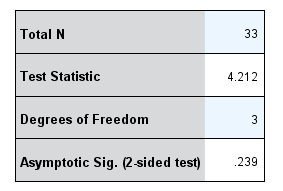

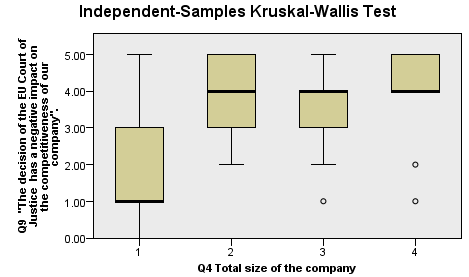


Figure E. Box-plot of results of Kruskal-Wallis test on response on Q9, categorized by total company size.

Testing any differences between company size and the (negative) effects on competition by means of a Kruskal-Wallis test, gives no significant (sig.=0,239) result (Figure E). However, the boxplot diagram shows a large difference between the modus of the results on statement Q9 of micro-sized companies compared to other-sized companies. The significance of this difference can be tested by grouping on the one hand micro-sized companies and on the other hand all larger firms. The outcomes of the corresponding Mann-Whitney U test are provided in Figure F.


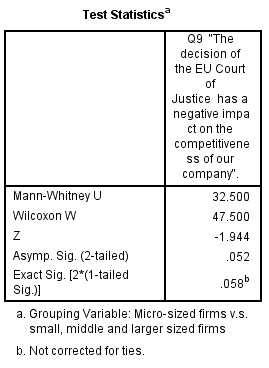

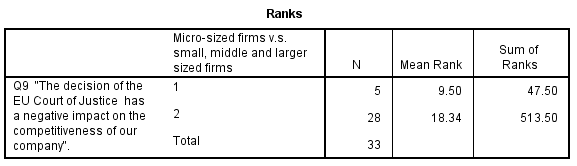


**Figure F.** Results of Mann-Whitney U test on results of Q9, grouped by micro-sized firms (1) and all larger firms (2)

The results of a Mann-Whitney U test give no significant result for the two-sided exact significance (0,058) and a significant result for testing one-sided (exact sig=0,029). This different effect in competitiveness for the micro-sized companies compared to larger sized companies might, as suggested by respondent 27, be due to the initial accessibility of CRISPR-Cas technique. Smaller, less capital-intensive companies are expected to have lower accessibility to this technique. Therefore, these companies might have some form of benefit from the ruling of the CJEU, as it equalizes the playing field in terms of use of technology. However, this level playing field has been pointed out by an EU market-oriented, large (>250 employees) company as well. Respondent 23 commented for example: “*Only a level playing field in the EU is crucial. End customers will get the products they want. If that is food without mutations, we're fine with that*”. This would however only be true for companies who compete within the EU-market. For EU-based companies who primarily compete on non-EU markets, the limiting factors of the CJEU ruling might experience larger negative effects in competitiveness. Also, note that two of the three respondents (5,22 and 29) who indicated that they are moving research outside the EU, had a non-EU main market. This relation between main-market and (negative) effect on competitiveness could however not be statistically proven. Based on the Kruskal-Wallis test, there did not appear to be a significant difference between the effects on competitiveness across the main market (2-sided asymptotic sig.: 0,170). Also, no significant result was found when differentiating between companies with main market within the EU and non-EU (2-sided asymptotic sig, 0,865). The latter result was however likely to have low significance due to the small number (N=5) of respondent having a non-EU main market. Besides the strong tendency towards agreement with the statement in results of the of Q9, three respondents (20, 29 and 31) specifically expressed their concerns about the competitiveness for the EU.

Based on Figure 1, panel A, the main text, the average results on Q9 appear to differ across seed sectors. This can however not be significantly proven, based on a Kruskal-Wallis test (Asymptotic sig= 0,066). Most striking is that the companies in the potato-sector on average disagree with the statement Q9, and therefore differ from all other sectors. This can also be statistically confirmed by performing a Mann-Whitney U test. With an exact significance of 0,007, the results appeared to be highly significant. Within the main submission, this result has been denoted in figure 1, panel B, with letter d. Further analysis among the characteristics, shows that by one exemption (Africa), all companies in the potato-sector appeared to have their main market within the EU. Also, the two respondents who pointed out the importance of a level-playing field (over the importance of the use of CRISPR-Cas), were both in the potato-sector. Furthermore, remember the significantly larger disagreement of the potato sector on Q7. On the other side of the spectrum, the companies in the Vegetable seeds sector, In Vitro and “other” sectors appear to agree the most to the statement on average. However, the distribution of the results on Q9 in these sectors do not differ significantly from others.

Besides testing the hypothesized negative effects of the CJEU Ruling, the results of the survey allow insights in two more factors that are related to the impact of the hypothesized effects of the ruling of the CJEU. First of all, the effects of the decision depend on the existence of equally important alternatives. When the substitutability of CRISPR-Cas technique is high, any limiting factors of the EU GMO procedure might be diminished. The results on the statement (Q10): “*There are equally important alternatives to CRISPR-Cas technology that are now being adopted within the company*.”, that tested the substitutability are provided in the table below (Table 9). According to these (raw) results, the substitutability of CRISPR-Cas appears to be rather low. Only 15,2% of the respondents agreed on having equally important technologies adopted within the company. Especially In Vitro Labs and companies who operate in multiple sectors (which all operate as In Vitro lab) appear to disagree with statement Q10.

Table 9. Results on statement Q10: "There are equally important alternatives to CRISPR-Cas technology that are now being adopted within the company."

When testing for all companies who operate as In Vitro lab (including Multi) compared to the remaining, the exact significance is 0,066, whilst the asymptotic significance is 0,044 (Figure G). However, considering that the sample size of the In Vitro labs is low (N=5), it is better justified to consider the exact significance, which means that the difference cannot said to be significant at 5% level.


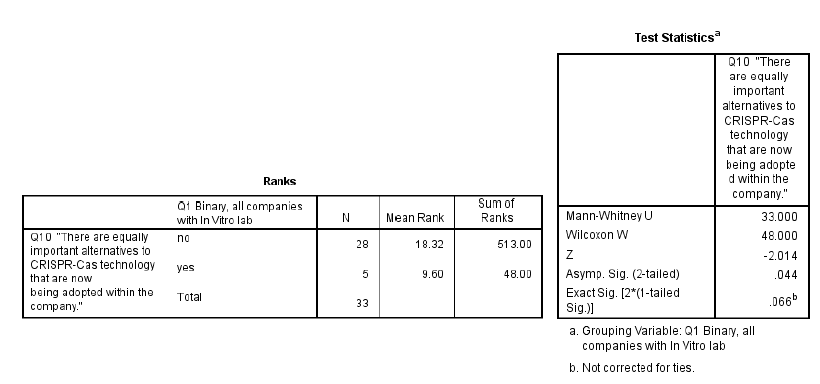


**Figure G.** Statistical output of Mann-Whitney U test on difference on results in Q10, binary grouped by companies with/without In Vitro lab.

The last two statements of the survey aimed to measure expectations of the companies for future legislation and the effect on the position of the Dutch plant breeding sector as a whole. The results on these questions are provided in Table 10.

Table 10. Distribution of responses on statement in Q11 (prospects on less stringent EU legislation in near future) and Q12 (losing leading position of Dutch plant breeding sector)

There appears to be a relatively positive attitude of the respondents towards the prospects of the strictness of the EU legislation around mutagenesis-based NPBTs. A majority of 60,6% of the respondents agreed to some degree with the statement in Q11: "*I believe precise mutagenetic technologies like CRISPR-Cas will be less stringently regulated within the EU in the near future (next 5 years)*". When looking at the differences in company-sizes (Figure 1a main text), large companies appear to have a relative pessimistic view, compared to smaller-sized companies. However, no significant differences were found (exact 2-sided sig=0,096). Among the seed-sectors (Figure 1b main text), companies who operate in the potato-sector are relatively pessimistic, compared to the other sector. However, also no significant difference was found (exact sig=0,308).

This general positivism about the development of legislation in the near future (within the next 5 years), is however somewhat contrary to the pessimism when asking about the position of the Dutch plant breeding sector as a world leader in the development of seeds for food production. The majority of 63,6% agreed with the statement (Q12) that the Dutch plant breeding sector will lose its leading position in the development of seeds for food production. Also, there seems to be a strong consensus among the different company-sizes in this expectation, based on Figure 1a main text. The same applies when differentiating the results by seed-sector (Figure 1b main text). However, companies in the potato-sector appear to be relatively optimistic in this case.

# Supplementary Data

Please see separate excel submission “Database Frontiers Perspective” for the raw data of the responses of the survey. Question 1 of the survey allowed for multiple answers, hence the multiple columns for this question. The additional comments have been included in the last column. Answers to the statements were based on a 5-point Likert scale, where 1 relates to *Strongly disagree* and 5 relates to *Strongly agree*. Answer 0 means the respondent indicated the answer as *Not Relevant.*

**Table 11.** Summary of survey results.


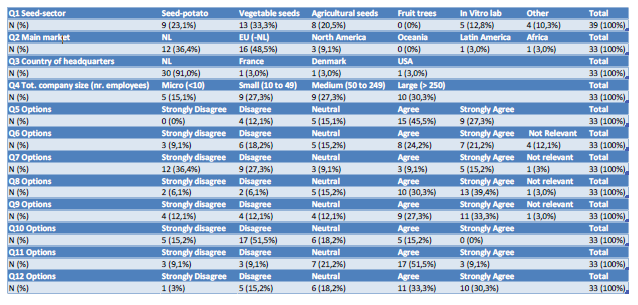


# Additional comments on survey

1. Respondent 5 on 18-2-2019 13:44:34

- “we will let do this research now outside Europe”

1. Respondent 11 on 12-2-2019 14:32:35

- “@Q6: CRIPR Cas technology COULD play an important role but it currently does not due to the GM status”

1. Respondent 18 on 2019-02-11 11:49:11

- “For question 2, there is only one answer possible. We are a global company, so all of markets , except Asia and Africa should be mentioned.”

1. Respondent 20 on 2019-02-11 10:58:54

- “as the Chines run much faster with OUR technology, without limitations the desicionof EU can be seen as a real strategic disaster . We create the Post docs and doctors in Wageningen to compete in the near future and become the world leaders we will have to obey in next generation.”

1. Respondent 22 on 2019-02-11 10:23:27

- “Research is moved outside EU”

1. Respondent 23 on 2019-02-11 9:29:19

- “Only a level playing field in the EU is crucial. End customers will get the products they want. If that is food without mutations, we're fine with that”

1. Respondent 27 on 2019-02-07 18:52:56

- “We have no possibilities to use these new tecniques and therefor can profit slightly from the EU ban on these techniques”

1. Respondent 29 on 2019-02-07 14:36:21

- “We will open up a US laboratory because of the ruling. Growth that could have taken place in the EU will now take place in the US. It's a ridiculous decision and will cost the EU dearly in innovation/ competitiveness and economic growth.”

1. Respondent 31 on 2019-02-07 10:17:03

- “Plant breeding is not a national item but a worldwide item. so if a techniek is available it wil be used and wenn it gives a benefit it will be spread out. So better to be involved with it then looking the orher way.”

# Supplementary figures


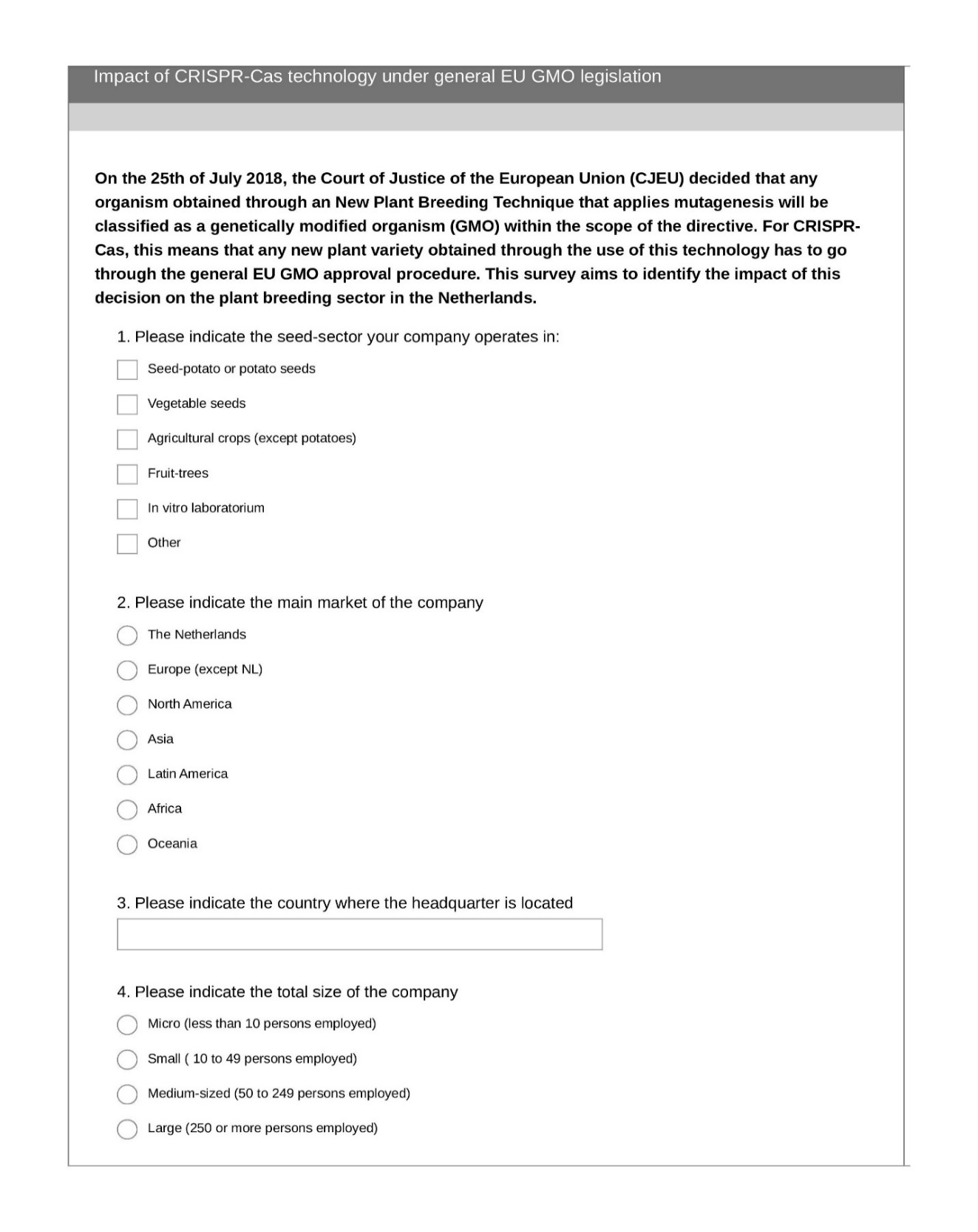


**Figure H.** First page of online survey on which quantitative section has been based.


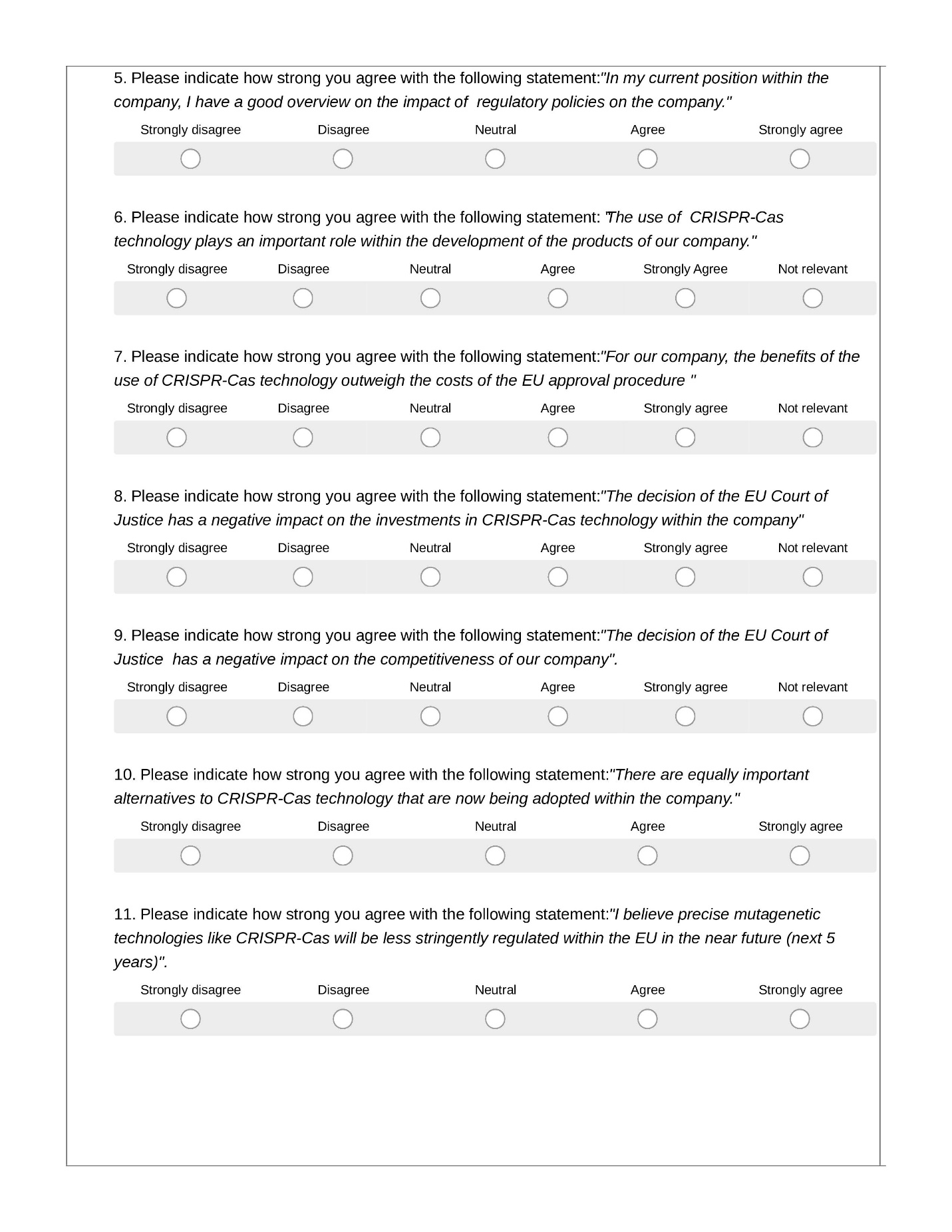
**Fig. Figure I.** Second page of online survey on which quantitative section has been based.


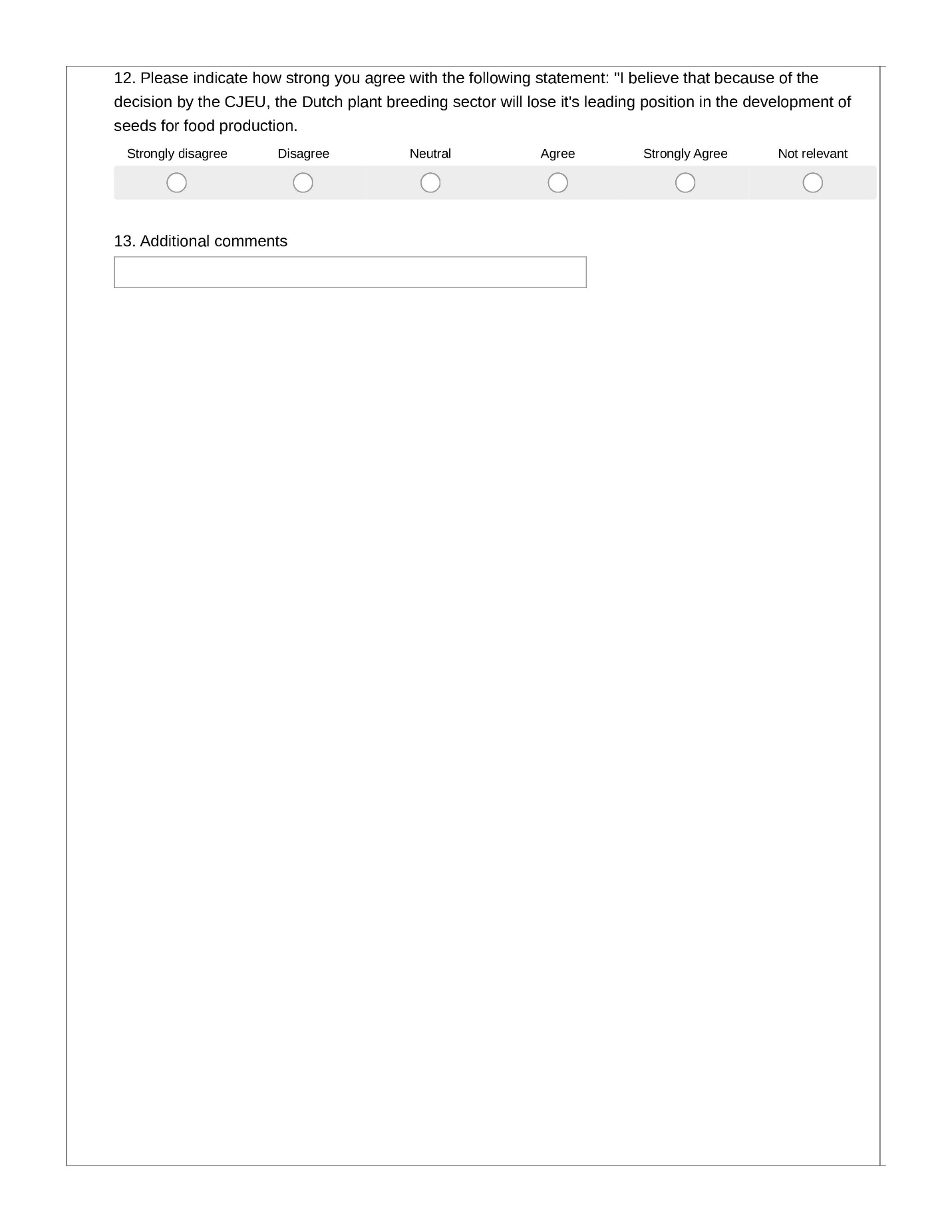
**Figure J.** Third page of online survey on which quantitative section has been based.
